# A unified approach and descriptor for the thermal expansion of two-dimensional transition metal dichalcogenide monolayers

**DOI:** 10.1126/sciadv.abo3783

**Published:** 2022-11-18

**Authors:** Yang Zhong, Lenan Zhang, Ji-Hoon Park, Samuel Cruz, Long Li, Liang Guo, Jing Kong, Evelyn N. Wang

**Affiliations:** ^1^ Department of Mechanical Engineering, Massachusetts Institute of Technology, Cambridge, MA 02139, USA.; ^2^ Department of Electrical Engineering and Computer Science, Massachusetts Institute of Technology, Cambridge, MA 02139, USA.; ^3^ Department of Mechanical and Energy Engineering, Southern University of Science and Technology, Shenzhen 518055, China.

## Abstract

Two-dimensional (2D) materials have enabled promising applications in modern miniaturized devices. However, device operation may lead to substantial temperature rise and thermal stress, resulting in device failure. To address such thermal challenges, the thermal expansion coefficient (TEC) needs to be well understood. Here, we characterize the in-plane TECs of transition metal dichalcogenide (TMD) monolayers and demonstrate superior accuracy using a three-substrate approach. Our measurements confirm the physical range of 2D monolayer TECs and, hence, address the more than two orders of magnitude discrepancy in literature. Moreover, we identify the thermochemical electronegativity difference of compositional elements as a descriptor, enabling the fast estimation of TECs for various TMD monolayers. Our work presents a unified approach and descriptor for the thermal expansion of TMD monolayers, which can serve as a guideline toward the rational design of reliable 2D devices.

## Teaser

A unified experimental approach reveals a physical descriptor for the thermal expansion coefficients of 2D materials.

## INTRODUCTION

Subnanometer thin two-dimensional (2D) materials and their heterogeneous integration have shown promise to meet the demands for the miniaturization of modern electronic and photonic devices (*1*, *2*). Strong interest in such atomically thin systems have been intensified owing to the recent discovery of direct bandgap (*3*), high carrier mobility (*4*), and strong spin-orbit coupling strength (*5*) in transition metal dichalcogenides (TMDs), which have enabled new frontiers for field-effect transistors (*6*), light-emitting diodes (*7*), and spintronics (*8*). To enable practical application of 2D devices, the thermal expansion coefficient (TEC, α) is one of the most important thermophysical properties of materials to be understood. On the one hand, the van der Waals (vdW) integration of 2D materials on dissimilar materials with TEC mismatch causes failure in epitaxial growth and material transfer, where the accurate characterization of TECs is critical (*9*, *10*). On the other hand, thermal management for miniaturized devices has become increasingly challenging because of the highly localized heat generation (*11*, *12*). The presence of ultrahigh thermal isolation across vdW interfaces limits heat dissipation (*13*, *14*) and can lead to substantial temperature rise (> 150°C) (*12*, *15*), which induces large thermal stresses that degrade device performance and accelerate device failure (*15*–*17*). Therefore, it is of critical importance to better understand the thermal expansion of 2D materials to optimize materials growth and transfer processes and improve the performance, reliability, and longevity of devices by minimizing thermal mismatch.

Nevertheless, the lack of in-depth understanding and inconsistent measurements of TECs have limited the ability for 2D materials to be used in practical applications. The TECs of 2D materials are difficult to directly measure since 2D materials are atomically thin and optically transparent. Previous works have used various metrologies to characterize TECs of 2D materials, such as scanning electron microscopy (*18*), transmission electron microscopy (*19*), X-ray diffraction (*20*), atomic force microscopy (*21*), and Raman spectroscopy (*16*, *22*–*26*). Despite tremendous efforts, only the TECs of a small portion of 2D materials have been studied in the past decade, and there exists a large discrepancy by more than two orders of magnitude for the same 2D material among the existing experimental characterizations (*24*, *26*–*28*), from ~10^−4^ K^−1^ (thermally expanding like liquids or polymers) to ~10^−7^ K^−1^ (as rigid as fused silica). The key challenge is that the substrate introduces strong effects of out-of-plane coupling (*29*, *30*) and nonuniform in-plane strain, while these experimental approaches typically neglect appropriate interpretation of such effects into measurements (*22*, *24*–*26*). Therefore, the understanding of TECs of 2D materials had heavily relied on theoretical modeling, such as molecular dynamics (MD) simulations (*31*, *32*) and first-principles calculations (*33*–*35*), yet without rigorous experimental support. However, first-principles calculations typically predict Grüneisen parameters that require detailed phonon-level calculations, which makes it difficult to directly translate these atomistic insights to guide practical material selection (*34*, *35*). It is thus an unresolved question what the physical range of TECs of 2D materials is and what dominant physics dictates their differences, making it challenging to understand 2D thermal expansion and optimize materials development and device design.

To address the above challenges, accurate experimental characterization that can decouple the substrate effect and extract the TEC of freestanding 2D materials is needed. Raman spectroscopy has been instrumental in characterizing thermal transport of miniaturized devices by capturing the response of phonons to temperature change, stress, and electric field (*30*, *36*–*41*). Recent studies have implemented Raman spectroscopy to characterize the TECs of graphene (*22*, *25*), hexagonal boron nitride (*40*), and MoS_2_ (*24*, *26*). However, conventional approaches with Raman spectroscopy rely on either empirical parameters or fitting enabled by theoretical calculations and fail to fully decouple the substrate effect (*22*, *24*–*26*, *40*). Therefore, these approaches provide highly inconsistent results, and the TECs of 2D materials remain largely unknown (*22*, *24*–*26*). Recently, we demonstrated a three-substrate approach that can accurately measure the TEC of MoS_2_ (*16*). The three-substrate approach combines independent Raman measurements on the three different substrates and decouples the contributions of temperature and substrate effects to the change of Raman peak positions. In this work, we show that we can accurately extract in-plane TECs of several TMD monolayers, MX_2_ (M = Mo, W; X = S, Se), using the three-substrate approach and address the large discrepancy reported in the literature by confirming the physical range of 2D TMD TECs. Our experimental characterization of the TECs of several TMD monolayers shows excellent agreement with the atomistic modeling. With insights gained from experiments, we confirm a much narrower physical range of in-plane TECs of TMD monolayers, from 5 × 10^−6^ to 1 × 10^−5^ K^−1^, as compared to the previously reported range, from ~10^−7^ to ~10^−4^ K^−1^ (*24*, *26*, *27*). Owing to the superior accuracy of our experimental characterization, the dependence of TECs on different compositional elements of TMDs within this narrow physical range can be well resolved. In particular, we show that the TEC of 2D TMDs linearly decreases with the dimensionless thermochemical electronegativity difference of compositional elements. This indicates that the thermochemical electronegativity difference can be used as a fundamental descriptor to elucidate the variations among TECs of TMDs and provide a new path to better understand 2D thermal expansion. To further guide material selections for the practical device design, we provide an empirical correlation between the TEC and the thermochemical electronegativity difference, which enables the rapid estimation of unknown TECs for various TMDs. Our work presents a unified approach and descriptor to characterize and understand the thermal expansion of monolayer TMDs, which can broadly affect fundamental understanding, materials growth, transfer, and device thermal management of 2D TMDs.

## RESULTS

### The coupled temperature and substrate effect

**Fig. 1.**
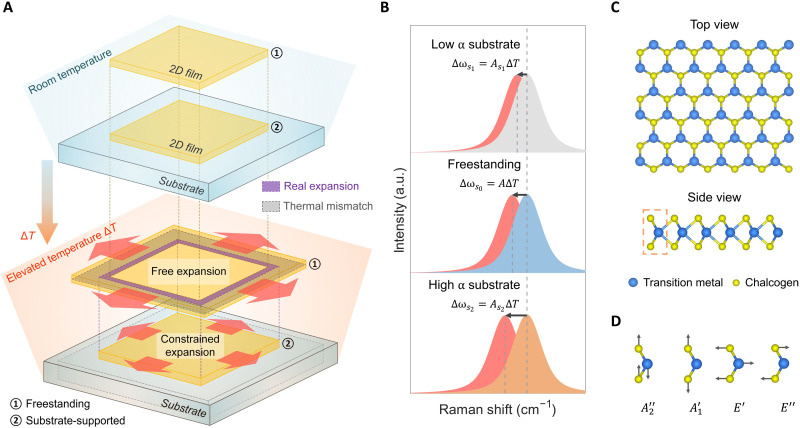
Understand thermal expansion via the coupled temperature and substrate effect on phonon modes. (**A**) Schematic of thermal expansion at room temperature and the elevated temperature. The 2D film is initially in thermal equilibrium with the substrate at room temperature. At the elevated temperature, the 2D film can freely expand if there are no substrates (①) but shows constrained expansion (real expansion) when it is supported by a low α substrate (②). Thermal mismatch, the difference between the free expansion and constrained expansion, induces large thermal stress leading to device failure. (**B**) Schematic Raman spectra of a 2D film on a low α substrate, a freestanding 2D film (suspended on microfabricated holes or slots), and a 2D film on a high α substrate at room temperature (represented in gray, blue, and orange shaded areas) and at the same elevated temperature (red shaded areas), showing the coupled temperature and substrate effect on Raman peak positions. The direction of arrows indicates the temperature increase, and the length of arrows represents the change of Raman peak positions of the 2D film, which also reveals the magnitude of the temperature coefficients, i.e., ∣Δω_*s*_2__∣ > ∣Δω_*s*_0__∣ > ∣Δω_*s*_1__∣ because of ∣*A*_*s*_2__∣ > ∣*A*∣ > ∣*A*_*s*_1__∣. (**C**) Top view and side view of the lattice structure of TMD monolayer that belongs to *D*_3*h*_ point group. Dashed rectangular box, unit cell of the lattice of monolayer TMD. (**D**) Phonon modes of monolayer TMD, MX_2_ (M = Mo, W; X = S, Se). The ${A\prime }_{1}$ and *E*′ modes are Raman active in the backscattering configuration for TMD monolayers. a.u., arbitrary units.

We elucidate the impact of temperature and substrate on a 2D film in Fig. 1A. As an example, we consider a 2D film supported by a bulk substrate with a smaller TEC. This 2D film is initially in thermal equilibrium with the substrate at room temperature. At the elevated temperature, both the 2D film and the substrate thermally expand. However, compared with the thermal expansion of a freestanding 2D film [① in Fig. 1A], the thermal expansion of the substrate-supported 2D film [② in Fig. 1A] is constrained because of thermal mismatch between the 2D film and the substrate. As a result, there is compressive thermal stress (σ_s_ < 0) within the 2D film, which can be described by the strain-stress relations

$$\varepsilon_{s}=\alpha \Delta T+\frac{1-\nu }{E}\sigma_{s}=\alpha_{s}\Delta T$$ (1)

where ε_s_ is the in-plane thermal strain within the 2D film when being supported by the substrate, α is the TEC of the 2D film, Δ*T* is the temperature rise, ν and *E* are the Poisson’s ratio and the Young’s modulus of the 2D film, σ_s_ is the in-plane thermal stress within the 2D film due to thermal mismatch, and α_s_ is the linear TEC of the substrate material. The relation holds valid when the thickness of the substrate (~mm) is much larger than that of the 2D film (~nm). For a highly thermal-expanding substrate, the 2D film will instead have tensile stress (σ_s_ > 0) with a temperature rise. The coupled temperature and substrate effect can be captured by Raman spectroscopy through (*16*, *42*)

$$\Delta \omega_{s}^{\left(n\right)}=A^{\left(n\right)}\Delta T+K^{\left(n\right)}\sigma_{s}$$ (2)

where Δω_s_ is the change of Raman peak position, *n* represents the Raman-active vibrational mode, *A*^(*n*)^ is the freestanding temperature coefficient, and *K*^(*n*)^ is the stress coefficient. Since σ_s_ is induced by Δ*T* (Eq. 1), the coupled effect on $\Delta \omega_{s}^{\left(n\right)}$ can also be represented as (see Materials and Methods for derivation)

$$\Delta \omega_{s}^{\left(n\right)}=A_{s}^{\left(n\right)}\Delta T$$ (3)

where $A_{s}^{\left(n\right)}$ is the temperature coefficient measured on substrate-supported 2D film, which includes the substrate effect. Note that the values of $A_{s}^{\left(n\right)}$ on different substrates are different. For a freestanding 2D film, there is no thermal stress upon a temperature rise, so $\Delta \omega_{s}^{\left(n\right)}=A^{\left(n\right)}\Delta T$.

Figure 1B illustrates the coupled effect of temperature and substrate with the schematic Raman spectra of the 2D film (Eq. 2). The Raman spectra of the 2D film on a low α substrate, freestanding 2D film, and 2D film on a high α substrate at room temperature are plotted in gray, blue, and orange shaded areas, respectively (Fig. 1B). The corresponding Raman spectra at the same elevated temperature are colored red, which shows a red shift due to phonon softening (indicated by the arrows). The length of arrows represents the change of Raman peak positions of the 2D film, which is determined by the substrate-dependent temperature coefficients $A_{s}^{\left(n\right)}$ according to Eq. 3. The freestanding 2D film can be prepared by suspending the sample on microfabricated holes or slots. It can be clearly seen that ∣Δω_*s*_2__∣ > ∣Δω_*s*_0__∣ > ∣Δω_*s*_1__∣ and ∣*A*_*s*_2__∣ > ∣*A*∣ > ∣*A*_*s*_1__∣ because compared to the freestanding 2D film, the low α substrate and the high α substrate give compressive and tensile thermal stresses, which turn out to reduce and amplify the temperature dependence of Raman peak position change, respectively (Eq. 3). In this work, we used monolayer TMD as a representative material. Figure 1C shows the top view and the side view of the lattice structure of the TMD monolayer, which belongs to the *D*_3*h*_ point group (*43*, *44*). Figure 1D shows the typical phonon modes of TMD monolayers. In the backscattering configuration, the out-of-plane mode ${A\prime }_{1}$ and the in-plane shear mode *E*′ can be detected by Raman spectroscopy (*16*, *45*).

### Extracting in-plane 2D TECs using the three-substrate approach

**Fig. 2.**
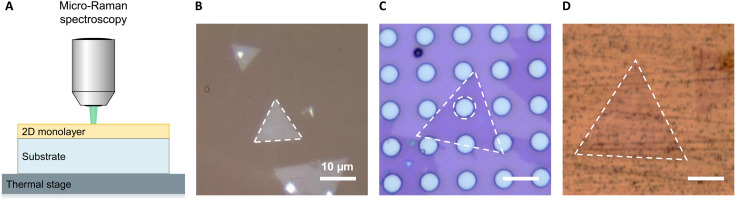
Three-substrate approach using micro-Raman spectroscopy. (**A**) Schematic of the experimental setup using micro-Raman spectroscopy. The CVD-grown TMD monolayers were transferred on three different substrates, (**B**) fused silica substrate (SiO_2_), (**C**) holey substrate with 5-μm-diameter hole patterns, and (**D**) copper substrate, separately. A thermal stage was interfaced with the substrate to control temperature for temperature-dependent Raman measurements. (B to D) ×100 magnification optical images of CVD-grown single-crystalline MoSe_2_ monolayer flakes on the three substrates. Dashed circle, 5-μm-diameter hole. Dashed triangles, single-crystalline monolayer flakes. Scale bars (B to D), 10 μm.

The above coupled temperature and substrate effect is one of the key bottlenecks to realize accurate measurement of TECs of 2D materials via conventional Raman spectroscopy–based approaches. However, the three-substrate approach can extract TECs by taking advantage of the strong temperature- and substrate-dependent Raman signatures (*16*). Figure 2A shows the concept of the three-substrate approach using micro-Raman spectroscopy with a laser spot diameter of ≈ 1 μm. The single-crystalline monolayer TMD flakes were transferred via a wet transfer process onto three different substrates: a fused silica substrate (SiO_2_, shown in Fig. 2B), a thermal oxide on silicon substrate with a micro-hole array (Fig. 2C), and a high-purity copper substrate (Fig. 2D), respectively. A thermal stage was interfaced with the bottom of the substrates to control the temperature for Raman measurements (see Materials and Methods for more information about sample preparation and Raman measurements). On the holey substrates, 2D monolayers were suspended over the hole area (Fig. 2B), where the diameter of the hole and the distance between adjacent holes are 5 μm. Because of the large aspect ratio of the hole diameter to the thickness of the 2D monolayer (≈ 5000), the compressive stress can be relaxed via buckling such that the 2D monolayers can be viewed as freestanding and can freely expand subject to the temperature increase (*16*, *42*, *46*). Figure 2 (B to D) shows ×100 magnification optical images of single-crystalline monolayer TMD flakes transferred on the fused silica substrate, the holey substrate, and the copper substrate, after being synthesized by chemical vapor deposition (CVD). The freestanding 2D monolayer flake, highlighted with the dashed triangles, was large enough (≈ 25 μm) to cover the hole area (≈ 5 μm diameter), marked with a dashed circle. MoSe_2_ was chosen as an example, and the optical images of other TMD monolayers characterized in this work, including monolayer WS_2_ and WSe_2_, can be found in section S1, where their monolayer nature was also confirmed by photoluminescence spectra (*47*–*50*). Temperature-dependent Raman measurements were performed on freestanding monolayer flakes to extract temperature coefficient *A*^(*n*)^ and substrate-supported monolayer flakes to extract temperature coefficients $A_{{\text{SiO}}_{2}}^{\left(n\right)}$ and $A_{\text{Cu}}^{\left(n\right)}$ for each 2D TMD (see Materials and Methods for the details of Raman measurements). With temperature coefficients characterized on the three substrates, the in-plane TECs of TMD monolayers are determined by (see Materials and Methods for the derivation of the three-substrate approach)

$$\alpha =\frac{(A_{\text{Cu}}^{\left(n\right)}-A^{\left(n\right)})\alpha_{{\text{SiO}}_{2}}-(A_{{\text{SiO}}_{2}}^{\left(n\right)}-A^{\left(n\right)})\alpha_{\text{Cu}}}{A_{\text{Cu}}^{\left(n\right)}-A_{{\text{SiO}}_{2}}^{\left(n\right)}},n={A\prime }_{1}\text{or}E\prime$$ (4)

where α_SiO_2__ = 0.55 × 10^−6^ K^−1^ and α_Cu_ = 16.5 × 10^−6^ K^−1^. As the theoretical framework is based on symmetry and perturbation analysis, the three-substrate approach is generally applicable to thin films and 2D materials, including those belonging to other symmetry groups (*16*).

**Fig. 3.**
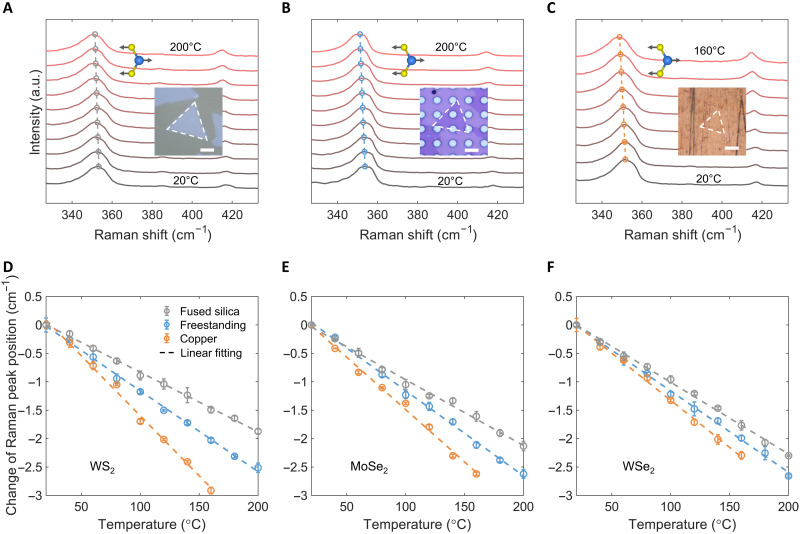
Temperature- and substrate-dependent Raman measurements on TMD monolayers. Raman spectra characterized at different temperatures for the *E*′ mode of (**A**) fused silica–supported WS_2_, (**B**) freestanding WS_2_, and (**C**) copper-supported WS_2_. The insets are the optical images of WS_2_ monolayers on the corresponding substrates. Scale bars, 10 μm. Dashed lines with circles, the change of Raman peak positions with temperature increase. The change of Raman peak positions, i.e., Raman peak positions subtracting the offset of their initial peak positions, as a function of temperature of (**D**) the *E*′ mode of WS_2_ monolayer, (**E**) the ${A\prime }_{1}$ mode of MoSe_2_ monolayer, and (**F**) the degenerate mode (assigned to either ${A\prime }_{1}$ or *E*′) of WSe_2_ monolayer. Raman measurements were performed on fused silica–supported, freestanding, and copper-supported TMD monolayers separately. The uncertainty was estimated as the SD of three independent Raman measurements. The temperature coefficients can be extracted by linearly fitting the change of Raman peak positions as a function of temperature.

Figure 3 (A and B) shows Raman spectra with temperatures increasing from 20° to 200°C with an interval of 20°C for the fused silica–supported and the freestanding WS_2_. The copper-supported WS_2_ was only heated up to 160°C for Raman measurements to avoid copper oxidation, as shown in Fig. 3C. Raman red shifts of the in-plane *E*′ mode were observed with increased temperature, as indicated by the dashed lines. Raman spectra of MoSe_2_ and WSe_2_ can be found in section S2. The residual stress from sample preparation may lead to variations of the initial Raman peak positions on different substrates. However, these variations only alter the absolute positions of Raman peaks and do not affect the temperature coefficients (*16*). Figure 3D depicts the change of Raman peak positions relative to their initial peak positions at 20°C as a function of temperature for the *E*′ mode of WS_2_ on the three substrates. The uncertainty of each Raman measurement was estimated as the standard deviation (SD) of three independent Raman measurements. The change of Raman peak positions with temperature rise has shown strong substrate dependence and good linear trends. The temperature coefficients were extracted from the slopes by linearly fitting (dashed lines of Fig. 3D) with *R*^2^ > 0.99. The *R*^2^ values of the linear fitting for all the temperature coefficients can be found in table S1. The linear relation between the change of Raman peak positions and temperature rise also indicates that there was no slip between the 2D monolayers and the substrates in the temperature range of our measurements due to the sufficiently strong vdW interactions (*16*, *46*). Specifically, the temperature coefficient of the freestanding WS_2_ monolayers *A*^*E*^′^^ was −0.0142 ± 0.0003 cm^−1^ K^−1^, where the uncertainty of temperature coefficient was estimated as the 95% confidence interval of linear regression. In comparison, the temperature coefficient of the WS_2_ monolayer transferred to the fused silica substrate $A_{{\text{SiO}}_{2}}^{E^{\prime }}$ was −0.0105 ± 0.0002 cm^−1^ K^−1^, whose magnitude was smaller than ∣*A*^*E*^′^^∣, indicating that the thermal mismatch between WS_2_ monolayers and the fused silica substrate induced compressive stress. However, the temperature coefficient of WS_2_ monolayer on the copper substrate $A_{\text{Cu}}^{E^{\prime }}$ was −0.0211 ± 0.0006 cm^−1^ K^−1^, whose magnitude was larger than ∣*A*^*E*′^∣ because of the tensile stress from the highly thermal-expanding copper substrate. Figure 3 (E and F) shows the temperature coefficient characterizations for the ${A\prime }_{1}$ modes of monolayer MoSe_2_ and WSe_2_. Note that the ${A\prime }_{1}$ mode and the *E*′ mode of monolayer WSe_2_ are almost degenerate at approximately 250 cm^−1^ (see the Raman spectra of WSe_2_ in section S2), and both were used in literature (*51*). We denote the detected mode of WSe_2_ as ${A\prime }_{1}$ mode to be consistent with that of MoSe_2_. The temperature coefficients of MoSe_2_ and WSe_2_ followed the same trend $|A_{\text{Cu}}^{\left(n\right)}|$ > ∣*A*^(*n*)^∣ > $|A_{{\text{SiO}}_{2}}^{\left(n\right)}|$. The results of the temperature coefficient characterization are summarized in Table 1.

**Table 1.** Temperature coefficients of freestanding, fused silica–supported, and copper-supported 2D TMD monolayers, as well as the corresponding in-plane TECs.

	***A*^(*n*)^ (cm^−1^ K^−1^)**	**$A_{\text{Si}O_{2}}^{\left(n\right)}$ (cm^−1^ K^−1^)**	**$A_{\text{Cu}}^{\left(n\right)}$ (cm^−1^ K^−1^)**	**α (×10^−6^ K^−1^)**
WS_2_	−0.0142 ± 0.0003	−0.0105 ± 0.0002	−0.0211 ± 0.0006	6.1 ± 0.6
MoSe_2_	−0.0151 ± 0.0003	−0.0116 ± 0.0003	−0.0184 ± 0.0005	8.8 ± 1.0
WSe_2_	−0.0143 ± 0.0002	−0.0126 ± 0.0002	−0.0166 ± 0.0004	7.3 ± 1.1

Incorporating the measured temperature coefficients into Eq. 4, the in-plane TEC of monolayer WS_2_ was (6.1 ± 0.6) × 10^−6^ K^−1^ for the *E*′ mode. In addition to the *E*′ mode, the ${A\prime }_{1}$ mode of WS_2_ at approximately 416 cm^−1^ was also detectable (Fig. 3, A to C). For this reason, we also extracted the TEC of WS_2_ monolayer from the ${A\prime }_{1}$ mode independently. The TEC characterized using the ${A\prime }_{1}$ mode of WS_2_ is (5.3 ± 1.7) × 10^−6^ K^−1^, which showed good consistency with the TEC of the *E*′ mode (see section S3 for the details). This result indicated that the three-substrate approach is a highly self-consistent approach to measure the TECs of 2D materials. The detailed uncertainty analysis of the three-substrate approach can be found in section S4. Since the three-substrate method relied on the calibration in the temperature range from 20° to 200°C, the good linear relationship between the change of Raman peak position and the temperature rise indicated that the TEC remained approximately constant within this temperature range. Considering that this temperature range is the typical operating range of 2D devices, our characterized TEC values are useful to guide practical design. We also performed MD simulation to confirm the insignificant temperature dependence of TECs of TMD monolayers from 20° to 200°C (see Materials and Methods and section S5 for more information). Only MS_2_ (M = Mo, W) monolayers were studied here as the force field used cannot well describe the mechanical properties of MSe_2_ (M = Mo, W) monolayers (*52*–*54*). The negligible temperature dependence of TECs from 20° to 200°C was also confirmed by density functional theory (DFT) calculations (*35*). The TECs determined from MD showed good agreement with our experiments. The TECs of TMDs characterized in this work are summarized in Table 1. We report the TEC of WS_2_ as (6.1 ± 0.6) × 10^−6^ K^−1^ with low experimental uncertainty because it was characterized from the shear mode *E*′, which is more sensitive to in-plane thermal stress.

### The physical range and descriptor for 2D TMD TEC

**Fig. 4.**
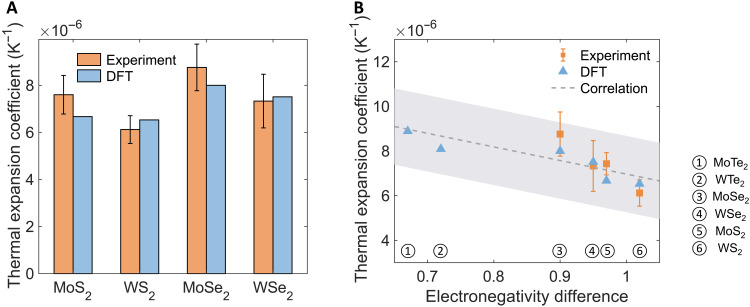
A unified approach and descriptor for the thermal expansion of monolayer TMDs. (**A**) Comparison of the in-plane TECs measured by the three-substrate approach and DFT calculations (*35*). The experimental TEC of the MoS_2_ monolayer is from (*16*). The experimental results and theoretical calculations show excellent agreement. The TECs of 2D TMD monolayers are on the order of 10^−6^ K^−1^ from experimental characterization and theoretical calculations. (**B**) The TECs of monolayer TMDs as a function of the dimensionless thermochemical electronegativity difference. The thermochemical electronegativity difference can serve as a good descriptor to elucidate the variations of TECs of the monolayer TMDs. Dashed line, the empirical linear correlation between α and Δχ_M − X_ by fitting combined experimental data and existing theoretical predictions. Gray region, the uncertainty band of 1 SD (σ = 1.7 × 10^−6^ K^−1^) for the empirical linear correlation.

To further confirm experimental accuracy, we compared the TECs obtained from the three-substrate approach with DFT calculations (*35*) in Fig. 4A and observed excellent agreement. Both experimental and theoretical results show that the TECs of TMD monolayers are on the order of 10^−6^ K^−1^. Our accurate measurements addressed the large discrepancies of 2D TMD TECs found in literature. Specifically, Hu *et al.* (*27*) performed a first-principles modeling–assisted experimental approach and showed that the in-plane TECs of TMD monolayers were ~10^−4^ K^−1^, which is comparable with the TECs of polymers and liquids and two orders of magnitude higher than typical semiconductors (~10^−6^ K^−1^) (*55*). Lin *et al.* (*24*) also studied 2D MoS_2_ using Raman spectroscopy without experimentally incorporating the substrate effect and reported the TEC to be approximately 5 × 10^−7^ K^−1^ at room temperature, which is comparable with the TECs of the most rigid materials like fused silica (*55*) and more than two orders of magnitude lower than the TEC given by Hu *et al.* (*27*). In addition, Anemone *et al.* (*28*) claimed that the natural bulk MoS_2_ lattice parameter was found to remain constant in a large temperature range, where they concluded that the TEC of MoS_2_ is zero. To elucidate the discrepancies of 2D TMD TECs due to inaccurate measurements, we tabulated TECs of various materials in table S3, showing a physical spectrum of thermal expansion regimes from liquids to solids. Since the mechanism of thermal expansion is governed by interatomic bond strength and thermal effects, the TECs of 2D materials with the same lattice structures should be within the similar range on the order of 10^−6^ K^−1^ above the Debye temperature. Therefore, through our accurate experimental measurements, we confirm that the TECs of 2D TMDs (from 5 × 10^−6^ to 1 × 10^−5^ K^−1^) fall into the same physical range as the TECs of common semiconductors (~10^−6^ K^−1^) (*55*). In general, the TECs of TMD monolayers are higher than the bulk values reported in the literature (*27*, *56*–*58*), which is attributed to the absence of interlayer vdW interactions (*16*). However, further experimental and theoretical efforts need to be pursued to not only provide accurate measurements of bulk TECs but also better understand the physical origin of the difference between 2D and bulk thermal expansion.

Owing to the highly accurate characterization, we can further understand the role of compositional elements in determining TECs of 2D TMDs. Within the physical range, our experiments show consistent compositional element dependence of TECs with DFT calculations (Fig. 4A). For example, the TEC of MoX_2_ is larger than that of WX_2_ (X = S, Se, Te), while the TEC of MS_2_ is smaller than that of MSe_2_ (M = Mo, W). With quasi-harmonic approximations, theoretical studies typically predict the TEC by calculating Grüneisen parameters for individual phonon modes from a thermodynamic point of view (*22*, *35*). Although these first-principles approaches can provide detailed information of various fundamental parameters, it is still difficult to construct a simple and clear physical picture to interpret these interesting trends and further guide materials development and selection. As the thermal expansion is the interplay of chemical bonding and thermal effects, the thermochemical nature of M–X bonds is crucial. Compared with the Grüneisen parameters, which require expensive computation, the electronegativity difference between the metal and chalcogen atoms is not only much more widely accessible but also a simpler fundamental parameter describing the polar nature of M–X bonds (*59*, *60*). Specifically, the concept of electronegativity was developed by Pauling in 1931 (*59*), describing the tendency of an atom of a given element to attract shared electrons in a covalent bond with a unit of eV^1/2^. A higher value of the electronegativity indicates that the element attracts the shared electrons more strongly. Tantardini and Oganov (*60*) further reformulated Pauling’s electronegativity with experimental dissociation energies, which leads to the dimensionless thermochemical electronegativity. Figure 4B shows that Tantardini and Oganov’s dimensionless thermochemical electronegativity difference Δχ_M−X_ between M and X atoms can well capture the variations in the TECs of monolayer TMDs, where a decreasing linear dependence between Δχ_M−X_ and TECs was observed. This trend is because a larger Δχ_M−X_ indicates a stronger polarity between M and X atoms. As a result, the material is stiffer, and its thermal expansion becomes lower (*35*, *61*, *62*). The validity of using Δχ_M−X_ to qualitatively describe the properties of M–X bonds was also confirmed in a study focused on the mechanical properties of atomically thin WX_2_ (*54*). We further show the strong dependence among the in-plane stiffness predicted using DFT calculations (*35*), electronegativity difference, and TECs of 2D TMD in section S7. The close relationship indicates that thermochemical electronegativity difference is a fundamental, intuitive, and simple descriptor to the TECs of TMD monolayers. Furthermore, since the value of the thermochemical electronegativity for each element is already available (*60*), the discovery of the linear dependence between α and Δχ_M−X_ can enable a rapid estimation of unknown TECs of other 2D TMD in practical applications without complex first-principles computations and experiments. Therefore, we suggest the following empirical correlation to estimate α using Δχ_M−X_ as a descriptor, by fitting our experimental data and existing theoretical predictions with a line (dashed line in Fig. 4B)

$$\alpha =k{\Delta \chi }_{M-X}+\beta$$ (5)

where *k* = −6.1 × 10^−6^ K^−1^ and β = 13.1 × 10^−6^ K^−1^. The *R*^2^ of the linear fitting is 0.6192, and the uncertainties of *k* and β, estimated as the 95% confidence intervals, were 1.7 × 10^−6^ and 1.5 × 10^−6^ K^−1^, respectively. The estimation uncertainty of the empirical correlation σ = 1.71 × 10^−6^ K^−1^ was evaluated as 1 SD of linear fitting (the gray uncertainty band in Fig. 4B).

## DISCUSSION

We performed a three-substrate approach to extract the in-plane TECs of 2D TMD monolayers based on micro-Raman spectroscopy. The purely experimental approach considered thermal mismatch and free expansion and was carried out by temperature-dependent Raman measurements on three substrates, including a holey substrate, a fused silica substrate, and a copper substrate, to decouple substrate and temperature effects. Our measurements show excellent agreement with atomistic simulation, which addresses the large discrepancy in the literature and confirms the physical range of in-plane TECs of 2D TMD monolayers (from 5 × 10^−6^ to 1 × 10^−5^ K^−1^) that was not well understood before. Owing to the high accuracy, this unified approach can even resolve the impact of elements on the TECs of TMD monolayers. We showed that the dimensionless thermochemical electronegativity difference can elucidate the variations among TECs of several TMDs monolayers and suggested an empirical correlation to rapidly estimate the TECs of various TMDs. Our work presents a unified approach and descriptor to characterize and understand the thermal expansion of 2D TMD monolayers, which can serve as a reference to optimize material growth and transfer processes, and improve the performance, reliability, and longevity of devices by minimizing thermal mismatch.

## MATERIALS AND METHODS

### Material growth

Monolayer WSe_2_ flakes were grown under low pressure by metal-organic CVD. Tungsten hexacarbonyl [W(CO)_6_, Sigma-Aldrich] and dimethyl selenide [(CH_3_)_2_Se, Sigma-Aldrich] were selected as precursors of W and Se, respectively, and were supplied in a gas phase into a 2.45-cm quartz tube furnace by the help of a bubbler system with Ar as a carrier gas. The WSe_2_ flakes were synthesized on a 300-nm-thick SiO_2_/Si wafer with the flow rate of 100 standard cubic centimeters per minute (sccm) of Ar, 1 sccm of H_2_, 0.3 sccm of W(CO)_6_, and 0.05 sccm of (CH_3_)_2_Se for 5 hours under the growth temperature of 420°C. After growth, the furnace heat was turned down, and the furnace was cooled down until it reached room temperature. The WS_2_ samples used in this work were purchased from 2Dsemiconductors USA and were CVD-grown on the c-cut sapphire. The MoSe_2_ samples were purchased from 6Carbon Technology and were grown by CVD on the 300-nm SiO_2_/Si wafer.

### Material transfer

The material transfer of TMDs was done via a poly(methyl methacrylate) (PMMA)–based wet transfer process. PMMA (A4, Micro Chem) was spin-coated on the SiO_2_/Si or sapphire substrate with TMD at 2500 rpm for 1 min. The spin-coated material stack was floated on 1 M KOH (for SiO_2_ substrate) or diluted HF (for sapphire substrate) solution for 1–2 hours to separate the PMMA film embedded with the TMD monolayer (PMMA/TMD) from the substrate. The PMMA/TMD stack was floated on deionized water overnight to remove any KOH or HF solution residue and then transferred onto the target substrates, including the holey substrate, the fused silica substrate (500 μm thick, double side polished, University Wafer), and the copper substrate (500 μm thick, 99.999% purity, American Elements). The sample was dried in on oven at 70°C for 10 min for good attachment. The remaining PMMA layer was removed by acetone.

### Substrate fabrication

The holey substrate was fabricated starting with a 500-μm-thick, 100-mm <100> Si wafer with a 500-nm layer of wet SiO_2_ (University Wafer). The wafer was first coated with a layer of hexamethyldisilazane (HMDS) in a vapor oven to promote adhesion of the photoresist. Then, SPR220-3 photoresist was spun on the wafer at 4000 rpm to yield a 3-μm layer of resist. A post-spin bake was performed at 115°C for 90 s. A Heidelberg MLA-150 direct-write photolithography tool was used to write the hole array onto the wafer in 1 cm by 1 cm sections. A post-exposure bake was performed at 115°C for 90 s. Then, the photoresist was developed using a Shipley Microposit MF CD-26 developer by submerging the wafer for 60 s. The wafer was rinsed and dried with N_2_. A post-develop bake was performed at 115°C for 90 s. Last, an STS inductively coupled plasma etching system was used to etch the thermal oxide layer through until it reached the Si/SiO_2_ interface, leaving behind a 500-nm-deep holey array substrate.

### Raman measurement

A Renishaw inVia Reflex confocal Raman microscope was used to collect Raman spectra. The Raman scattering was excited using a 532-nm wavelength diode laser and focused by a 100× numerical aperture = 0.75 microscope objective. The laser power was maintained below 0.5 mW to avoid excessive laser heating. The samples were heated by a temperature control stage (Linkam THMS600). Raman spectra were measured with temperature from 20° to 200°C with an interval of 20°C for both the suspended and fused silica–supported samples. The flakes on the copper substrate were only heated up to 160°C to avoid notable copper oxidation. The Raman peak positions were obtained by fitting experimental data to the Voigt profile (*16*, *38*). The uncertainty of each Raman peak position was estimated from the SD of three independent measurements.

### Description of the three-substrate approach

The strain-stress relations of the 2D film supported by the fused silica and copper substrates can be expressed as

$$\varepsilon_{{\text{SiO}}_{2}}=\alpha \Delta T+\frac{1-\nu }{E}\sigma_{{\text{SiO}}_{2}}=\alpha_{{\text{SiO}}_{2}}\Delta T$$ (6)

$$\varepsilon_{\text{Cu}}=\alpha \Delta T+\frac{1-\nu }{E}\sigma_{\text{Cu}}=\alpha_{\text{Cu}}\Delta T$$ (7)

where ε_SiO_2__ and ε_Cu_ are the in-plane strains of the 2D films supported by the fused silica and copper substrates, respectively. σ_SiO_2__ and σ_Cu_ are the corresponding in-plane thermal stresses in the 2D films induced by the substrates. Plugging Eqs. 6 and 7 into Eq. 2, the coupled temperature and substrate effect on the change of Raman peak positions through thermal stresses σ_SiO_2__ and σ_Cu_ can be explicitly written in the form of Eq. 3

$$\Delta \omega_{{\text{SiO}}_{2}}^{\left(n\right)}=(\frac{K^{\left(n\right)}(\alpha_{{\text{SiO}}_{2}}-\alpha )E}{1-\nu }+A^{\left(n\right)})\Delta T=A_{{\text{SiO}}_{2}}^{\left(n\right)}\Delta T,n={A\prime }_{1}\;\text{or}\;E\prime$$ (8)

$$\Delta \omega_{\text{Cu}}^{\left(n\right)}=(\frac{K^{\left(n\right)}(\alpha_{\text{Cu}}-\alpha )E}{1-\nu }+A^{\left(n\right)})\Delta T=A_{\text{Cu}}^{\left(n\right)}\Delta T,n={A\prime }_{1}\text{or}\;E\prime$$ (9)

Equations 8 and 9 indicate that the stress coefficient *K*^(*n*)^ and the constant term $\frac{E}{1-\nu }$ can be eliminated, and the TEC α can be related to temperature coefficients of the Raman measurements $A_{{\text{SiO}}_{2}}^{\left(n\right)}$, $A_{\text{Cu}}^{\left(n\right)}$, and *A*^(*n*)^ using Eq. 4.

### MD simulation

Classical MD simulation was performed to calculate the TEC using the LAMMPS package (*63*). The freestanding TMD monolayer was approximately 50 nm long along each in-plane direction. The intramolecular interactions were modeled by Stillinger-Weber potentials (*64*). Periodic boundary conditions were applied to the in-plane directions, while no restriction was applied to the cross-plane direction. With a timestep of 0.5 fs, the monolayer was initially relaxed in a constant pressure (zero) condition for 3 ns, followed by 2 ns used to record the free expanding length. The lattice constants of the material were estimated at different temperatures so that it can be expressed as a function of temperature. The linear TEC α*_i_*(*T*) can be calculated using

$$\alpha_{i}=\frac{1}{l}\frac{\partial l}{\partial T}$$ (10)

where *l* is the lattice constant of the TMD monolayer, and *i* indicates the in-plane direction of the simulation domain. The reported TEC using MD simulation is the average TEC $\bar{\alpha }$ since no strong anisotropy was observed along the two in-plane directions. More details can be found in section S5.

## Supplementary Materials

### This PDF file includes:

Sections S1 to S7 Figs. S1 to S10 Tables S1 to S3

## References

[1] K. S. Novoselov, A. Mishchenko, A. Carvalho, A. H. Castro Neto, 2D materials and van der Waals heterostructures. Science 353, aac9439 (2016). 27471306 10.1126/science.aac9439

[2] P. C. Shen, C. Su, Y. Lin, A. S. Chou, C. C. Cheng, J. H. Park, M. H. Chiu, A. Y. Lu, H. L. Tang, M. M. Tavakoli, G. Pitner, X. Ji, Z. Cai, N. Mao, J. Wang, V. Tung, J. Li, J. Bokor, A. Zettl, C. I. Wu, T. Palacios, L. J. Li, J. Kong, Ultralow contact resistance between semimetal and monolayer semiconductors. Nature 593, 211–217 (2021). 33981050 10.1038/s41586-021-03472-9

[3] Y. Zhang, T. R. Chang, B. Zhou, Y. T. Cui, H. Yan, Z. Liu, F. Schmitt, J. Lee, R. Moore, Y. Chen, H. Lin, H. T. Jeng, S. K. Mo, Z. Hussain, A. Bansil, Z. X. Shen, Direct observation of the transition from indirect to direct bandgap in atomically thin epitaxial MoSe_2_. Nat. Nanotechnol. 9, 111–115 (2014). 24362235 10.1038/nnano.2013.277

[4] D. Xiao, G. Bin Liu, W. Feng, X. Xu, W. Yao, Coupled spin and valley physics in monolayers of MoS_2_ and other group-VI dichalcogenides. Phys. Rev. Lett. 108, 196802 (2012). 23003071 10.1103/PhysRevLett.108.196802

[5] S.-H. Gong, F. Alpeggiani, B. Sciacca, E. C. Garnett, L. Kuipers, Nanoscale chiral valley-photon interface through optical spin-orbit coupling. Science 359, 443–447 (2018). 29371466 10.1126/science.aan8010

[6] M. L. Chen, X. Sun, H. Liu, H. Wang, Q. Zhu, S. Wang, H. Du, B. Dong, J. Zhang, Y. Sun, S. Qiu, T. Alava, S. Liu, D. M. Sun, Z. Han, A FinFET with one atomic layer channel. Nat. Commun. 11, 1205 (2020). 32139679 10.1038/s41467-020-15096-0 PMC7058032

[7] J. Gu, B. Chakraborty, M. Khatoniar, V. M. Menon, A room-temperature polariton light-emitting diode based on monolayer WS_2_. Nat. Nanotechnol. 14, 1024–1028 (2019). 31548689 10.1038/s41565-019-0543-6

[8] J. F. Sierra, J. Fabian, R. K. Kawakami, S. Roche, S. O. Valenzuela, Van der Waals heterostructures for spintronics and opto-spintronics. Nat. Nanotechnol. 16, 856–868 (2021). 34282312 10.1038/s41565-021-00936-x

[9] H. Kum, D. Lee, W. Kong, H. Kim, Y. Park, Y. Kim, Y. Baek, S. H. Bae, K. Lee, J. Kim, Epitaxial growth and layer-transfer techniques for heterogeneous integration of materials for electronic and photonic devices. Nat. Electron. 2, 439–450 (2019).

[10] G. H. Ahn, M. Amani, H. Rasool, D. H. Lien, J. P. Mastandrea, J. W. Ager, M. Dubey, D. C. Chrzan, A. M. Minor, A. Javey, Strain-engineered growth of two-dimensional materials. Nat. Commun. 8, 608 (2017). 28931806 10.1038/s41467-017-00516-5 PMC5606995

[11] D. G. Cahill, P. V. Braun, G. Chen, D. R. Clarke, S. Fan, K. E. Goodson, P. Keblinski, W. P. King, G. D. Mahan, A. Majumdar, H. J. Maris, S. R. Phillpot, E. Pop, L. Shi, Nanoscale thermal transport. II. 2003–2012. Appl. Phys. Rev. 1, 011305 (2014).

[12] E. Yalon, C. J. McClellan, K. K. H. Smithe, M. Muñoz Rojo, R. L. Xu, S. V. Suryavanshi, A. J. Gabourie, C. M. Neumann, F. Xiong, A. B. Farimani, E. Pop, Energy dissipation in monolayer MoS_2_ electronics. Nano Lett. 17, 3429–3433 (2017). 28388845 10.1021/acs.nanolett.7b00252

[13] S. Vaziri, E. Yalon, M. M. Rojo, S. V. Suryavanshi, H. Zhang, C. J. McClellan, C. S. Bailey, K. K. H. Smithe, A. J. Gabourie, V. Chen, S. Deshmukh, L. Bendersky, A. V. Davydov, E. Pop, Ultrahigh thermal isolation across heterogeneously layered two-dimensional materials. Sci. Adv. 5, eaax1325 (2019). 31453337 10.1126/sciadv.aax1325 PMC6697438

[14] L. Zhang, Y. Zhong, X. Qian, Q. Song, J. Zhou, L. Li, L. Guo, G. Chen, E. N. Wang, Toward optimal heat transfer of 2D–3D heterostructures via van der Waals binding effects. ACS Appl. Mater. Interfaces 13, 46055–46064 (2021). 34529424 10.1021/acsami.1c08131

[15] C. Foy, L. Zhang, M. E. Trusheim, K. R. Bagnall, M. Walsh, E. N. Wang, D. R. Englund, Wide-field magnetic field and temperature imaging using nanoscale quantum sensors. ACS Appl. Mater. Interfaces 12, 26525–26533 (2020). 32321237 10.1021/acsami.0c01545

[16] L. Zhang, Z. Lu, Y. Song, L. Zhao, B. Bhatia, K. R. Bagnall, E. N. Wang, Thermal expansion coefficient of monolayer molybdenum disulfide using micro-Raman spectroscopy. Nano Lett. 19, 4745–4751 (2019). 31184905 10.1021/acs.nanolett.9b01829

[17] R. Roldán, A. Castellanos-Gomez, E. Cappelluti, F. Guinea, Strain engineering in semiconducting two-dimensional crystals. J. Phys. Condens. Matter 27, 313201 (2015). 26199038 10.1088/0953-8984/27/31/313201

[18] W. Bao, F. Miao, Z. Chen, H. Zhang, W. Jang, C. Dames, C. N. Lau, Controlled ripple texturing of suspended graphene and ultrathin graphite membranes. Nat. Nanotechnol. 4, 562–566 (2009). 19734927 10.1038/nnano.2009.191

[19] E. Kano, M. Malac, M. Hayashida, Substrate and contamination effects on the thermal expansion coefficient of suspended graphene measured by electron diffraction. Carbon 163, 324–332 (2020).

[20] K. Akikubo, T. Kurahashi, S. Kawaguchi, M. Tachibana, Thermal expansion measurements of nano-graphite using high-temperature X-ray diffraction. Carbon 169, 307–311 (2020).

[21] G. López-Polín, M. Ortega, J. G. Vilhena, I. Alda, J. Gomez-Herrero, P. A. Serena, C. Gomez-Navarro, R. Pérez, Tailoring the thermal expansion of graphene via controlled defect creation. Carbon 116, 670–677 (2017).

[22] D. Yoon, Y.-W. Son, H. Cheong, Negative thermal expansion coefficient of graphene measured by Raman spectroscopy. Nano Lett. 11, 3227–3231 (2011). 21728349 10.1021/nl201488g

[23] Q. Feng, D. Wei, Y. Su, Z. Zhou, F. Wang, C. Tian, Study of thermal expansion coefficient of graphene via Raman micro-spectroscopy: Revisited. Small 17, 2006146 (2021). 10.1002/smll.202006146 33634590

[24] Z. Lin, W. Liu, S. Tian, K. Zhu, Y. Huang, Y. Yang, Thermal expansion coefficient of few-layer MoS_2_ studied by temperature-dependent Raman spectroscopy. Sci. Rep. 11, 7037 (2021). 33782514 10.1038/s41598-021-86479-6 PMC8007611

[25] S. Tian, Y. Yang, Z. Liu, C. Wang, R. Pan, C. Gu, J. Li, Temperature-dependent Raman investigation on suspended graphene: Contribution from thermal expansion coefficient mismatch between graphene and substrate. Carbon 104, 27–32 (2016).

[26] D. J. Late, S. N. Shirodkar, U. V. Waghmare, V. P. Dravid, C. N. R. Rao, Thermal expansion, anharmonicity and temperature-dependent Raman spectra of single- and few-layer MoSe_2_ and WSe_2_. ChemPhysChem 15, 1592–1598 (2014). 24692405 10.1002/cphc.201400020

[27] X. Hu, P. Yasaei, J. Jokisaari, S. Öǧüt, A. Salehi-Khojin, R. F. Klie, Mapping thermal expansion coefficients in freestanding 2D materials at the nanometer scale. Phys. Rev. Lett. 120, 055902 (2018). 29481159 10.1103/PhysRevLett.120.055902

[28] G. Anemone, A. Al Taleb, A. Castellanos-Gomez, D. Farías, Experimental determination of thermal expansion of natural MoS_2_. 2D Mater. 5, 035015 (2018).

[29] B. Qiu, X. Ruan, Reduction of spectral phonon relaxation times from suspended to supported graphene. Appl. Phys. Lett. 100, 193101 (2012).

[30] W. Cai, A. L. Moore, Y. Zhu, X. Li, S. Chen, L. Shi, R. S. Ruoff, Thermal transport in suspended and supported monolayer graphene grown by chemical vapor deposition. Nano Lett. 10, 1645–1651 (2010). 20405895 10.1021/nl9041966

[31] İ. Demiroğlu, Y. Karaaslan, T. Kocabaş, M. Keçeli, Á. Vázquez-Mayagoitia, C. Sevik, Computation of the thermal expansion coefficient of graphene with gaussian approximation potentials. J. Phys. Chem. C 125, 14409–14415 (2021).

[32] S. Thomas, K. M. Ajith, S. Chandra, M. C. Valsakumar, Temperature dependent structural properties and bending rigidity of pristine and defective hexagonal boron nitride. J. Phys. Condens. Matter 27, 315302 (2015). 26190799 10.1088/0953-8984/27/31/315302

[33] C. Sevik, Assessment on lattice thermal properties of two-dimensional honeycomb structures: Graphene, h-BN, h-MoS_2_, and h-MoSe_2_. Phys. Rev. B 89, 035422 (2014).

[34] N. Mounet, N. Marzari, First-principles determination of the structural, vibrational and thermodynamic properties of diamond, graphite, and derivatives. Phys. Rev. B 71, 205214 (2005).

[35] Z. Y. Wang, Y. L. Zhou, X. Q. Wang, F. Wang, Q. Sun, Z. X. Guo, Y. Jia, Effects of in-plane stiffness and charge transfer on thermal expansion of monolayer transition metal dichalcogenide. Chinese Phys. B 24, 026501 (2015).

[36] S. Chen, A. L. Moore, W. Cai, J. W. Suk, J. An, C. Mishra, C. Amos, C. W. Magnuson, J. Kang, L. Shi, R. S. Ruoff, Raman measurements of thermal transport in suspended monolayer graphene of variable sizes in vacuum and gaseous environments. ACS Nano 5, 321–328 (2011). 21162551 10.1021/nn102915x

[37] S. Choi, E. Heller, D. Dorsey, R. Vetury, S. Graham, Analysis of the residual stress distribution in AlGaN/GaN high electron mobility transistors. J. Appl. Phys. 113, 093510 (2013).

[38] K. R. Bagnall, E. A. Moore, S. C. Badescu, L. Zhang, E. N. Wang, Simultaneous measurement of temperature, stress, and electric field in GaN HEMTs with micro-Raman spectroscopy. Rev. Sci. Instrum. 88, 113111 (2017). 29195348 10.1063/1.5010225

[39] E. Yalon, Ö. B. Aslan, K. K. H. Smithe, C. J. McClellan, S. V. Suryavanshi, F. Xiong, A. Sood, C. M. Neumann, X. Xu, K. E. Goodson, T. F. Heinz, E. Pop, Temperature-dependent thermal boundary conductance of monolayer MoS_2_ by Raman thermometry. ACS Appl. Mater. Interfaces 9, 43013–43020 (2017). 29053241 10.1021/acsami.7b11641

[40] Q. Cai, D. Scullion, W. Gan, A. Falin, S. Zhang, K. Watanabe, T. Taniguchi, Y. Chen, E. J. G. Santos, L. H. Li, High thermal conductivity of high-quality monolayer boron nitride and its thermal expansion. Sci. Adv. 5, eaav0129 (2019). 31187056 10.1126/sciadv.aav0129 PMC6555632

[41] Z. Han, X. Yang, S. E. Sullivan, T. Feng, L. Shi, W. Li, X. Ruan, Raman linewidth contributions from four-phonon and electron-phonon interactions in graphene. Phys. Rev. Lett. 128, 045901 (2022). 35148139 10.1103/PhysRevLett.128.045901

[42] S. Huang, Y. Chen, Z. Luo, X. Xu, Temperature and strain effects in micro-Raman thermometry for measuring in-plane thermal conductivity of thin films. Nanoscale Microscale Thermophys. Eng. 25, 91–100 (2021).

[43] S. Huang, L. Liang, X. Ling, A. A. Puretzky, D. B. Geohegan, B. G. Sumpter, J. Kong, V. Meunier, M. S. Dresselhaus, Low-frequency interlayer Raman modes to probe interface of twisted bilayer MoS_2_. Nano Lett. 16, 1435–1444 (2016). 26797083 10.1021/acs.nanolett.5b05015

[44] Y. Cai, J. Lan, G. Zhang, Y. W. Zhang, Lattice vibrational modes and phonon thermal conductivity of monolayer MoS_2_. Phys. Rev. B 89, 035438 (2014).

[45] S. Sahoo, A. P. S. Gaur, M. Ahmadi, M. J. F. Guinel, R. S. Katiyar, Temperature-dependent Raman studies and thermal conductivity of few-layer MoS_2_. J. Phys. Chem. C 117, 9042–9047 (2013).

[46] Z. Luo, J. Tian, S. Huang, M. Srinivasan, J. Maassen, Y. P. Chen, X. Xu, Large enhancement of thermal conductivity and lorenz number in topological insulator thin films. ACS Nano 12, 1120–1127 (2018). 29361229 10.1021/acsnano.7b06430

[47] P. Tonndorf, R. Schmidt, P. Böttger, X. Zhang, J. Börner, A. Liebig, M. Albrecht, C. Kloc, O. Gordan, D. R. T. Zahn, S. M. de Vasconcellos, R. Bratschitsch, Photoluminescence emission and Raman response of monolayer MoS_2_, MoSe_2_, and WSe_2_. Opt. Express 21, 4908–4916 (2013). 23482024 10.1364/OE.21.004908

[48] M. Yang, X. Cheng, Y. Li, Y. Ren, M. Liu, Z. Qi, Anharmonicity of monolayer MoS_2_, MoSe_2_, and WSe_2_: A Raman study under high pressure and elevated temperature. Appl. Phys. Lett. 110, 093108 (2017).

[49] H. Zeng, G. Bin Liu, J. Dai, Y. Yan, B. Zhu, R. He, L. Xie, S. Xu, X. Chen, W. Yao, X. Cui, Optical signature of symmetry variations and spin-valley coupling in atomically thin tungsten dichalcogenides. Sci. Rep. 3, 1608 (2013). 23575911 10.1038/srep01608 PMC3622914

[50] X. Fan, W. Zheng, H. Liu, X. Zhuang, P. Fan, Y. Gong, H. Li, X. Wu, Y. Jiang, X. Zhu, Q. Zhang, H. Zhou, W. Hu, X. Wang, X. Duan, A. Pan, Nonlinear photoluminescence in monolayer WS_2_: Parabolic emission and excitation fluence-dependent recombination dynamics. Nanoscale 9, 7235–7241 (2017). 28513703 10.1039/c7nr01345k

[51] H. Terrones, E. Del Corro, S. Feng, J. M. Poumirol, D. Rhodes, D. Smirnov, N. R. Pradhan, Z. Lin, M. A. T. Nguyen, A. L. Elías, T. E. Mallouk, L. Balicas, M. A. Pimenta, M. Terrones, New first order Raman-active modes in few layered transition metal dichalcogenides. Sci. Rep. 4, 4215 (2014). 24572993 10.1038/srep04215 PMC5379439

[52] W. Ding, D. Han, J. Zhang, X. Wang, Mechanical responses of WSe_2_ monolayers: A molecular dynamics study. Mater. Res. Express 6, 085071 (2019).

[53] X. Wang, Y. Hong, M. Wang, G. Xin, Y. Yue, J. Zhang, Mechanical properties of molybdenum diselenide revealed by molecular dynamics simulation and support vector machine. Phys. Chem. Chem. Phys. 21, 9159–9167 (2019). 30801579 10.1039/c8cp07881e

[54] A. Falin, M. Holwill, H. Lv, W. Gan, J. Cheng, R. Zhang, D. Qian, M. R. Barnett, E. J. G. Santos, K. S. Novoselov, T. Tao, X. Wu, L. H. Li, Mechanical properties of atomically thin tungsten dichalcogenides: WS_2_, WSe_2_, and WTe_2_. ACS Nano 15, 2600–2610 (2021). 33503379 10.1021/acsnano.0c07430

[55] M. Kaviany, *Essentials of Heat Transfer: Principles, Materials, and Applications* (Cambridge Univ. Press, ed. 1, 2011).

[56] L. H. Brixner, X-ray study and thermoelectric properties of the W*_x_*Ta_1−*x*_Se_2_ system. J. Electrochem. Soc. 110, 289 (1963).

[57] Y. Ding, B. Xiao, Thermal expansion tensors, Grüneisen parameters and phonon velocities of bulk MT_2_ (M = W and Mo; T = S and Se) from first principles calculations. RSC Adv. 5, 18391–18400 (2015).

[58] S. H. El-Mahalawy, B. L. Evans, The thermal expansion of 2H-MoS_2_, 2*H*-MoSe_2_ and 2*H*-WSe_2_ between 20 and 800°C. J. Appl. Cryst. 9, 403–406 (1976).

[59] L. Pauling, The nature of the chemical bond. IV. The energy of single bonds and the relative electronegativity of atoms. J. Am. Chem. Soc. 54, 3570–3582 (1932).

[60] C. Tantardini, A. R. Oganov, Thermochemical electronegativities of the elements. Nat. Commun. 12, 2087 (2021). 33828104 10.1038/s41467-021-22429-0 PMC8027013

[61] E. Le Bourhis, *Glass Mechanics and Technology* (Wiley-VCH, ed. 1, 2007).

[62] C. Kittel, H. Kroemer, *Thermal Physics* (W. H. Freeman, ed. 2, 1980).

[63] S. Plimpton, Fast parallel algorithms for short-range molecular dynamics. J. Comput. Phys. 117, 1–19 (1995).

[64] J. W. Jiang, Parametrization of Stillinger-Weber potential based on valence force field model: Application to single-layer MoS_2_ and black phosphorus. Nanotechnology 26, 315706 (2015). 26184637 10.1088/0957-4484/26/31/315706

