# Supplementary material for: A unified approach and descriptor for the thermal expansion of two-dimensional transition metal dichalcogenide monolayers
Source: Sci Adv. 2022 Nov 18;8(46):eabo3783. doi: 10.1126/sciadv.abo3783 (PMC9674296; doi:10.1126/sciadv.abo3783)
Supplement: Supplementary file 1 — Sections S1 to S7 Figs. S1 to S10 Tables S1 to S3 [file sciadv.abo3783_sm.pdf]

Science Advances
AAAS

Supplementary Materials for

A unified approach and descriptor for the thermal expansion of two-dimensional transition metal dichalcogenide monolayers

Yang Zhong *et al.*

Corresponding author: Liang Guo, guol3@sustech.edu.cn; Jing Kong, jingkong@mit.edu; Evelyn N. Wang, enwang@mit.edu

Sci. Adv. **8**, eabo3783 (2022)
DOI: 10.1126/sciadv.abo3783

This PDF file includes:

Sections S1 to S7
Figs. S1 to S10
Tables S1 to S3

Section S1. TMD monolayer preparation and optical characterization

Three transition metal dichalcogenide (TMD) monolayers were characterized in this work. Monolayer WS_2 samples were purchased from 2Dsemiconductors USA and were grown by chemical vapor deposition (CVD) on the c-cut sapphire. The $MoSe_2$ samples were purchased from 6Carbon Technology and were grown by CVD on the 300 nm SiO_2/Si wafer. The WSe_2 flakes were grown by metal-organic chemical vapor deposition (MOCVD) as described in the main text. The material transfer of TMDs was done via a poly(methyl methacrylate) (PMMA) based wet transfer process onto the target substrates, including the holey substrate, the fused silica substrate (double side polished, University Wafer), and the copper substrate (99.999% purity, American Elements). The procedures of material transfer and the holey substrate fabrication were discussed in the main text. The monolayers flakes were characterized by optical microscope (Figs. 2B-D, S1 and S2) and their monolayer nature was also confirmed using the photoluminescence (PL) spectroscopy. Figure S3 shows the PL spectra of WS_2, WSe_2 and $MoSe_2$ monolayers collected using 532 nm, 532 nm, and 488 nm laser excitation, respectively. Three PL spectra were centered at 2.01 eV, 1.63 eV, and 1.55 eV, which agreed well with the literature (*47–50*).

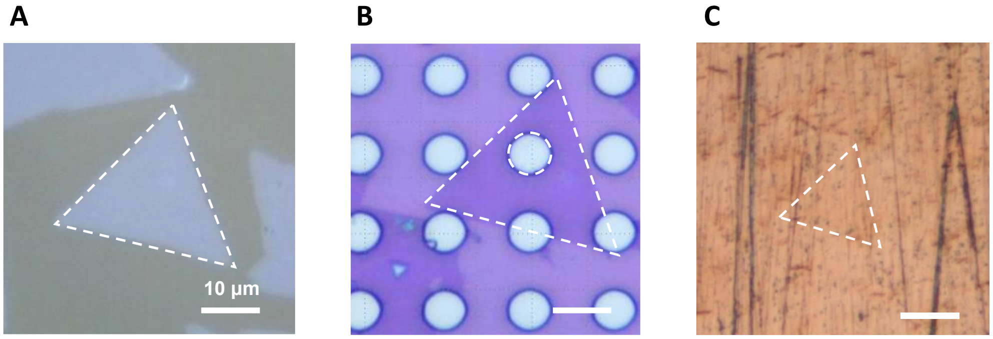

Fig. S1. Optical images of WS_2 on three substrates. 100× magnification optical images of CVD-grown WS_2 monolayers transferred on (**A**) fused silica substrate, (**B**) holey substrate with 5 µm diameter hole patterns, and (**C**) copper substrate, separately. Dashed circle: 5 µm diameter hole. Dashed triangles: single-crystalline monolayer WS_2 flakes. Scale bars represent 10 µm.

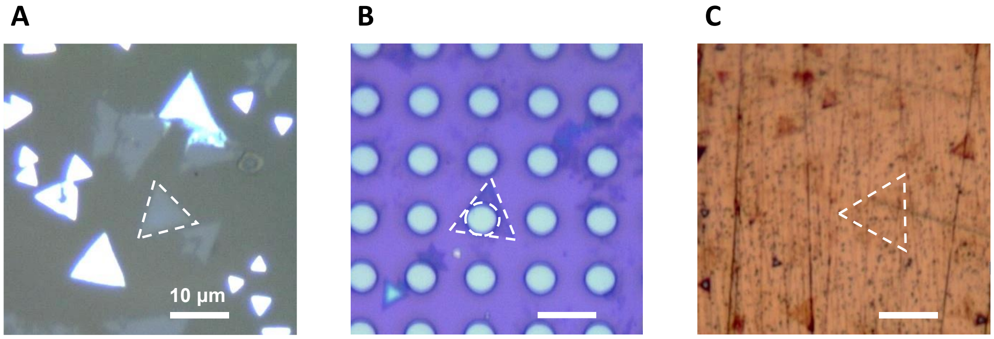

Fig. S2. Optical images of WSe_2 on three substrates. 100× magnification optical images of MOCVD-grown WSe_2 monolayers transferred on (**A**) fused silica substrate, (**B**) holey substrate with 5 µm diameter hole patterns, and (**C**) copper substrate, separately. Dashed circle: 5 µm diameter hole. Dashed triangles: single-crystalline monolayer WSe_2 flakes. Scale bars represent 10 µm.

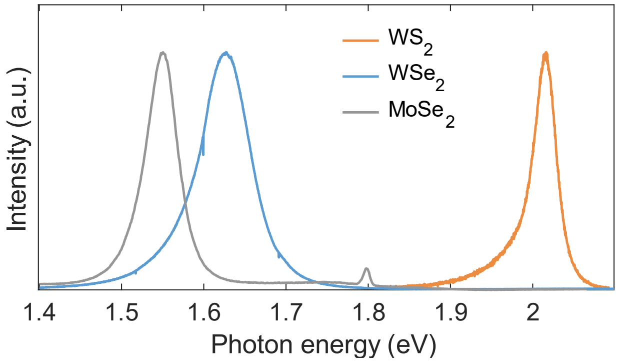

Fig. S3. Photoluminescence spectra of TMD monolayers in this work. Strong photoluminescent signals of WS_2, WSe_2 and, $MoSe_2$ monolayers were observed. The PL spectra of WS_2, WSe_2 and $MoSe_2$ monolayers were normalized and centered at 2.01 eV, 1.63 eV, and 1.55 eV, respectively.

Section S2. Temperature- and substrate-dependent Raman measurements

We performed the temperature-dependent Raman measurements on free-standing monolayer flakes to extract temperature coefficient $A^{(n)}$, and substrate-supported monolayer flakes to extract temperature coefficients $A^{(n)}_{\mathrm{SiO_2}}$ and $A^{(n)}_{\mathrm{Cu}}$. A Renishaw inVia Reflex confocal Raman microscope was used to collect Raman spectra. The Raman scattering was excited using a 532 nm wavelength diode laser and focused by a 100× NA=0.75 microscope objective. The laser power was maintained below 0.5 mW to avoid excessive laser heating.

Raman measurements for the characterization of the E' mode of monolayer WS_2 can be found in the main text (Fig. 3A-C), and the characterization of the A_1' mode can be found in Section S3. Figure S4 (A and B) shows Raman spectra with temperatures increasing from 20 to 200 °C with an interval 20 °C for the fused silica supported and the free-standing $MoSe_2$. Figure S4C shows Raman measurements for the copper supported $MoSe_2$, which was heated up to 160 °C to avoid copper oxidation. Raman redshifts of the A_1' mode were observed with increased temperature and the peak positions follow good linear trends (Fig. 3E). Compared to the A_1' mode, the E' mode of $MoSe_2$ was weak, especially for the free-standing $MoSe_2$, and cannot be well-resolved, which was also observed in other literature (*47*, *48*). As for monolayer WSe_2, only one characteristic peak was detected because the A_1' mode and the E' mode were almost degenerate at approximately 250 cm^{-1} and both notations were used in the literature (*47*, *48*, *51*). Here, we report the detected mode as A_1' mode to be consistent with that of $MoSe_2$. Figure S5 shows temperature-dependent Raman spectra for the WSe_2 on the three substrates with the same temperature range used for WS_2 and $MoSe_2$. Raman redshifts were observed with temperature rise, and the peak positions follow good linear trends with the increase of temperature (Fig. 3F).

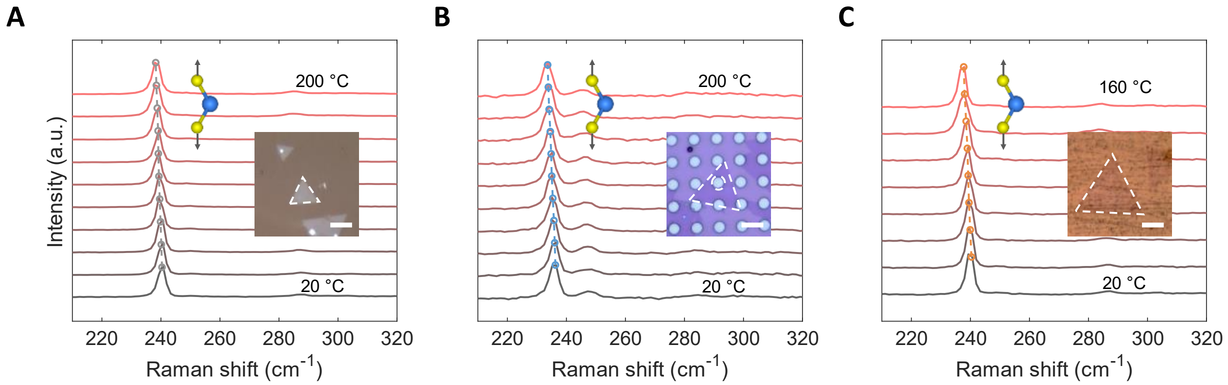

Fig. S4. Temperature- and substrate-dependent Raman measurements for $MoSe_2$ monolayers. Raman spectra characterized at different temperatures for the A_1' mode of (**A**) fused silica supported $MoSe_2$, (**B**) free-standing $MoSe_2$, and (**C**) copper supported $MoSe_2$. The insets are the optical images of $MoSe_2$ monolayers on the corresponding substrates. Scale bars represent 10 μm. Dashed lines with circles: change of Raman peak positions.

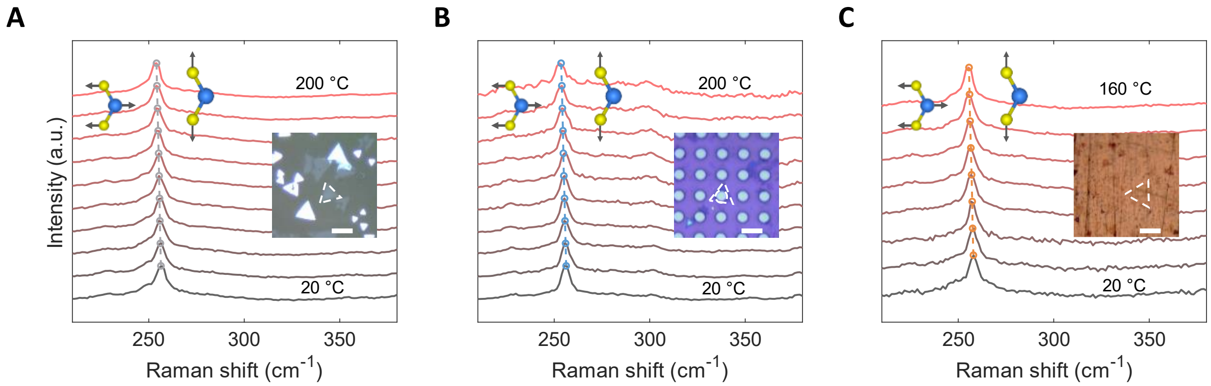

Fig. S5. Temperature- and substrate-dependent Raman measurements for WSe_2 monolayers. Raman spectra characterized at different temperatures for the degenerate mode of (**A**) fused silica supported WSe_2, (**B**) free-standing WSe_2, and (**C**) copper supported WSe_2. The insets are the optical images of WSe_2 monolayers on the corresponding substrates. Scale bars represent 10 μm. Dashed lines with circles: change of Raman peak positions.

Section S3. Characterization of the A_1' mode of monolayer WS_2

Figure S6 shows Raman spectra with temperatures increasing from 20 to 200 °C for the fused silica supported and the free-standing WS_2, and to 160 °C for the copper supported WS_2, with an interval 20 °C. Raman redshifts were observed with increased temperature, and the peak positions follow good linear trends. Figure S7 shows the change of Raman peak positions as a function of temperature for WS_2 monolayers on the three substrates, where strong substrate dependence and good linear trends can be observed. The temperature coefficient of the free-standing WS_2 monolayer $A^{A_1'}$ was −0.0139 ± 0.0002 cm^{-1} K^{-1}. The temperature coefficient of the WS_2 monolayer on the fused silica substrate $A_{SiO_2}^{A_1'}$ was −0.0130 ± 0.0003 cm^{-1} K^{-1}, whose magnitude was small than $\left|A^{A_1'}\right|$, indicating that there was thermal mismatch between WS_2 flakes and the fused silica substrate, inducing compressive stress. The temperature coefficient of WS_2 monolayer on the copper substrate $A_{Cu}^{A_1'}$ was −0.0160 ± 0.0005 cm^{-1} K^{-1}, whose magnitude was larger than $\left|A^{A_1'}\right|$ due to the tensile stress from the highly thermal-expanding copper substrate. The relation followed $\left|A_{Cu}^{A_1'}\right| > \left|A^{A_1'}\right| > \left|A_{SiO_2}^{A_1'}\right|$, which was same as $\left|A_{Cu}^{E'}\right| > \left|A^{E'}\right| > \left|A_{SiO_2}^{E'}\right|$ as discussed in the main text. The in-plane thermal expansion coefficient (TEC) of monolayer WS_2 is (5.3 ± 1.7) × 10^{-6} K^{-1} using the A_1' mode. In comparison, the TEC characterized using the E' mode of WS_2 is (6.1 ± 0.6) × 10^{-6} K^{-1}. The good agreement between these two independent characterizations demonstrates the consistency of the three-substrate approach. The uncertainty determined from the A_1' mode was larger than that from the E' mode because the values of $A_{Cu}^{A_1'}$ and $A_{SiO_2}^{A_1'}$ were very close to each other. We show in the uncertainty analysis that the uncertainty is inversely proportional to $A_{Cu}^{(n)} - A_{SiO_2}^{(n)}$. Therefore, the closer the two temperature coefficients are, the larger the uncertainty will be.

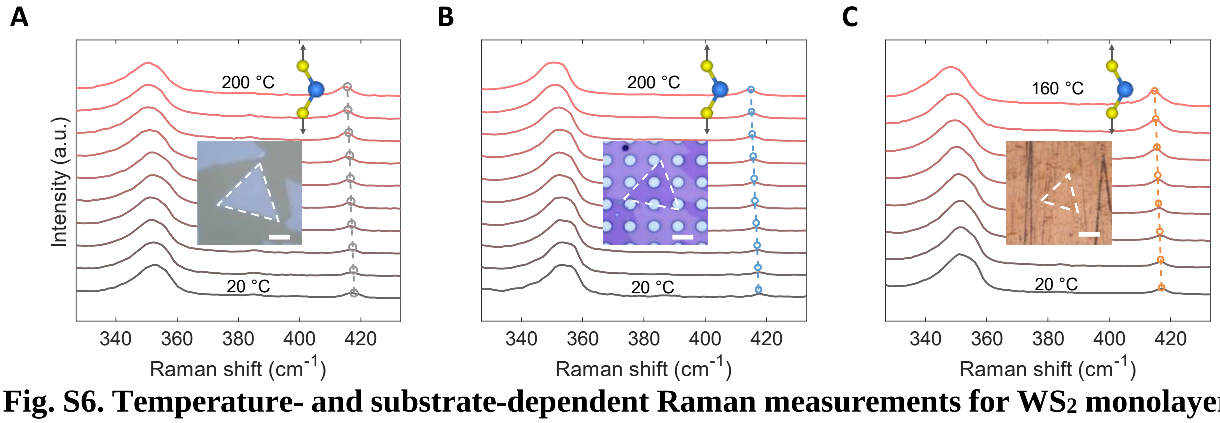

Fig. S6. Temperature- and substrate-dependent Raman measurements for WS_2 monolayers. Raman spectra characterized at different temperatures for the A_1' mode of (**A**) fused silica supported WS_2, (**B**) free-standing WS_2, and (**C**) copper supported WS_2. The insets are the optical images of WS_2 monolayers on the corresponding substrates. Scale bars represent 10 µm. Dashed lines with circles: change of Raman peak positions.

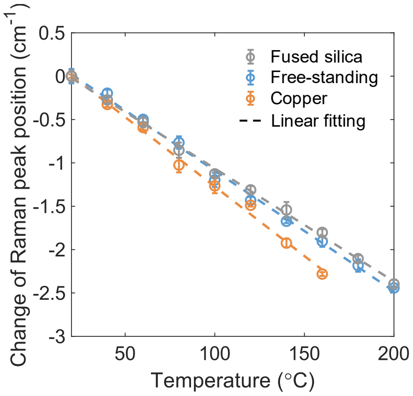

Fig. S7. Temperature coefficient characterization on the A_1' mode of WS_2 monolayers. The change of Raman peak positions as a function of temperature of the A_1' mode of WS_2 monolayer. Raman measurements were performed on fused silica supported, free-standing, and copper supported TMD monolayers separately. The temperature coefficients were extracted from linearly fitting the change of Raman peak positions to the temperature.

Section S4. Uncertainty analysis of the three-substrate approach

The temperature coefficients were extracted from the slopes by linearly fitting the change of the Raman peak positions with temperature rise. The R^2 values of the linear fitting for temperature coefficients are summarized in Table S1, which are all close to unity, indicating good linear trends. We adopted a similar uncertainty estimation approach used in previous Raman studies, where the dominant source of error of the Raman measurement was considered as the random error of multiple measurements (*16*, *38*). The uncertainties of temperature coefficients were estimated as the 95% confidence interval of linear regression. According to the uncertainty propagation principle, the total uncertainty of the measured TEC can be expressed as,

$$\delta\alpha = \sqrt{\left(\frac{\partial\alpha}{\partial A_{\mathrm{Cu}}^{(n)}}\delta A_{\mathrm{Cu}}^{(n)}\right)^2 + \left(\frac{\partial\alpha}{\partial A_{\mathrm{SiO_2}}^{(n)}}\delta A_{\mathrm{SiO_2}}^{(n)}\right)^2 + \left(\frac{\partial\alpha}{\partial A^{(n)}}\delta A^{(n)}\right)^2 + \left(\frac{\partial\alpha}{\partial \alpha_{\mathrm{Cu}}}\delta \alpha_{\mathrm{Cu}}\right)^2 + \left(\frac{\partial\alpha}{\partial \alpha_{\mathrm{SiO_2}}}\delta \alpha_{\mathrm{SiO_2}}\right)^2} \quad \text{(S1)}$$

where $\delta A_{\mathrm{Cu}}^{(n)}$, $\delta A_{\mathrm{SiO_2}}^{(n)}$, and $\delta A^{(n)}$ were obtained through the linear fitting, and $\delta\alpha_{\mathrm{Cu}}$ and $\delta\alpha_{\mathrm{SiO_2}}$ were from material vendors (American Elements and University Wafer). The partial differences shown in Eq. S1 are:

$$\frac{\partial\alpha}{\partial A_{\mathrm{Cu}}^{(n)}} = \frac{\alpha_{\mathrm{SiO_2}}\left(A_{\mathrm{Cu}}^{(n)} - A_{\mathrm{SiO_2}}^{(n)}\right) - \left(\left(A_{\mathrm{Cu}}^{(n)} - A^{(n)}\right)\alpha_{\mathrm{SiO_2}} - \left(A_{\mathrm{SiO_2}}^{(n)} - A^{(n)}\right)\alpha_{\mathrm{Cu}}\right)}{\left(A_{\mathrm{Cu}}^{(n)} - A_{\mathrm{SiO_2}}^{(n)}\right)^2} \quad \text{(S2)}$$

$$\frac{\partial\alpha}{\partial A_{\mathrm{SiO_2}}^{(n)}} = \frac{-\alpha_{Cu}\left(A_{\mathrm{Cu}}^{(n)} - A_{\mathrm{SiO_2}}^{(n)}\right) + \left(\left(A_{\mathrm{Cu}}^{(n)} - A^{(n)}\right)\alpha_{\mathrm{SiO_2}} - \left(A_{\mathrm{SiO_2}}^{(n)} - A^{(n)}\right)\alpha_{\mathrm{Cu}}\right)}{\left(A_{\mathrm{Cu}}^{(n)} - A_{\mathrm{SiO_2}}^{(n)}\right)^2} \quad \text{(S3)}$$

$$\frac{\partial\alpha}{\partial A^{(n)}} = \frac{\alpha_{\mathrm{Cu}} - \alpha_{\mathrm{SiO_2}}}{A_{\mathrm{Cu}}^{(n)} - A_{\mathrm{SiO_2}}^{(n)}} \quad \text{(S4)}$$

$$\frac{\partial\alpha}{\partial \alpha_{\mathrm{SiO_2}}} = \frac{A_{\mathrm{Cu}}^{(n)} - A^{(n)}}{A_{\mathrm{Cu}}^{(n)} - A_{\mathrm{SiO_2}}^{(n)}} \quad \text{(S5)}$$

$$\frac{\partial\alpha}{\partial \alpha_{\mathrm{Cu}}} = -\frac{A_{\mathrm{SiO_2}}^{(n)} - A^{(n)}}{A_{\mathrm{Cu}}^{(n)} - A_{\mathrm{SiO_2}}^{(n)}} \quad \text{(S6)}$$

The partial derivatives of uncertainties are all inversely proportional to $A_{\mathrm{Cu}}^{(n)} - A_{\mathrm{SiO_2}}^{(n)}$. The closer the two extracted temperature coefficients are, the larger the resulting uncertainty will be. The uncertainty of each quantity is listed in Table S2. In the temperature coefficient characterization of the A_1' mode of WS_2 monolayers, $A_{\mathrm{Cu}}^{A_1'}$ and $A_{\mathrm{SiO_2}}^{A_1'}$ are very close to each other, leading to a larger uncertainty as compared to the uncertainty for the E' mode.

Table S1. R^2 values of the linear fitting for the temperature coefficients.

R^2	$A_{\mathrm{SiO_2}}^{A_1'}$	$A_{\mathrm{SiO_2}}^{E'}$	$A_{\mathrm{Cu}}^{A_1'}$	$A_{\mathrm{Cu}}^{E'}$	$A^{A_1'}$	$A^{E'}$
WS_2	0.9979	0.9979	0.9953	0.9948	0.9960	0.9975
$MoSe_2$	0.9940	-	0.9947	-	0.9972	-
WSe_2	0.9970	-	0.9972	-	0.9979	-

Table S2. Uncertainties in the analysis.

	$\delta A_{\mathrm{SiO_2}}^{A_1'}$ (cm^{-1} K^{-1})	$\delta A_{\mathrm{SiO_2}}^{E'}$ (cm^{-1} K^{-1})	$\delta A_{\mathrm{Cu}}^{A_1'}$ (cm^{-1} K^{-1})	$\delta A_{\mathrm{Cu}}^{E'}$ (cm^{-1} K^{-1})	$\delta A^{A_1'}$ (cm^{-1} K^{-1})	$\delta A^{E'}$ (cm^{-1} K^{-1})	$\delta \alpha_{\mathrm{Cu}}$ (×10^{-7} K^{-1})	$\delta \alpha_{\mathrm{SiO_2}}$ (×10^{-7} K^{-1})
WS_2	0.0003	0.0002	0.0005	0.0006	0.0002	0.0003	1	0.2
$MoSe_2$	0.0003	-	0.0005	-	0.0003	-		
WSe_2	0.0002	-	0.0004	-	0.0002	-		

Section S5. Calculating TEC from molecular dynamics simulation

Classical molecular dynamics (MD) simulations were performed to calculate the thermal expansion coefficients of free-standing MoS_2 and WS_2 monolayers using the LAMMPS package (*63*). Figure S8A shows the in-plane lattice structure of TMD monolayers. The constructed MoS_2 monolayer was 49.92 × 43.23 nm^2 large, while the dimensions of WS_2 monolayer were 50.08 nm × 43.37 nm^2. The intramolecular interactions were modeled by Stillinger-Weber potentials (*64*). Periodic boundary conditions were applied to the in-plane directions, and no restriction was applied to the cross-plane direction. The monolayer was relaxed in a zero pressure and temperature condition for 3 ns, followed by 2 ns used to record the free-expanding lengths along in-plane directions, with a timestep of 0.5 fs throughout the simulation. The lattice constants were estimated at different temperature so that it can be expressed as a function of temperature. Figure S8 (B and C) shows the lattice constants of monolayer MoS_2 and WS_2 as a function of temperature calculated from MD simulation. We fitted the lattice constant with a 4th order polynomial function to (the dashed line) to obtain an analytical expression of lattice constant as a function of temperature, $l = l(T)$. The linear TEC $\alpha_i(T)$ can thus be calculated using,

$$\alpha_i = \frac{1}{l}\frac{\partial l}{\partial T}, i = x, y, \tag{S8}$$

where l is the lattice constant of the TMD monolayer, and i indicates the in-plane direction of the simulation domain, i.e., armchair and zigzag. Since no strong anisotropy was observed along the two in-plane directions (less than 5% for MoS_2, less than 1% for WS_2), we show in Fig. S9 the average TECs of MoS_2 and WS_2 monolayers as $\bar{\alpha} = \frac{\alpha_x + \alpha_y}{2}$. Note that we performed Raman measurements in the temperature range from 20 °C to 200 °C, which is also the temperature range that a device is usually operating at (*12*). Our results show that within this temperature range, the changes of α_{MoS_2} and α_{WS_2} are 24.2% and 12.8%, respectively. At T_m = 110 °C, $\bar{\alpha}_{MoS2}(T_m)$ = 7.32 × 10^{-6} K^{-1} and $\bar{\alpha}_{WS2}(T_m)$ = 5.59 × 10^{-6} K^{-1}, which agree reasonably well with the experimental measurements and first-principles calculations (*35*).

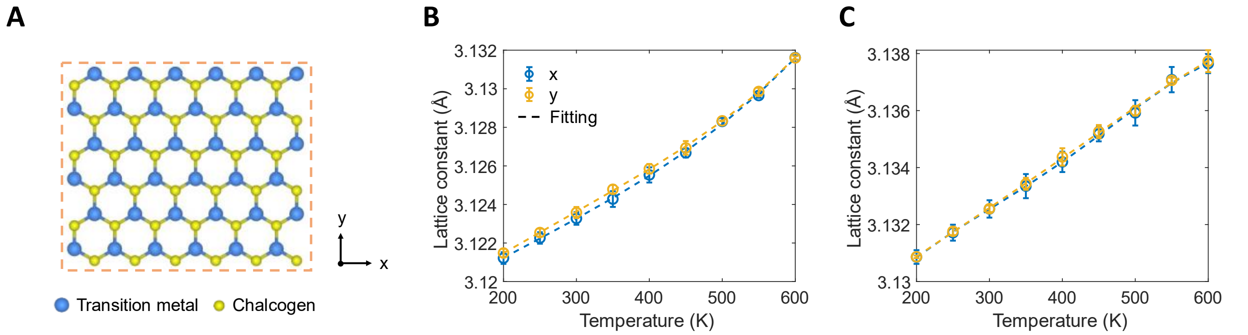

Fig. S8. Calculation of lattice constant using MD simulation. (**A**) The simulation domain of MD simulation. The calculation of lattice constant was based on the calculation of two in-plane directions. Dashed rectangular box: periodic boundary conditions applied in the simulation. Lattice constants of free-standing (**B**) MoS_2 and (**C**) WS_2 monolayers as function of temperature in MD simulation. The uncertainty of the lattice constant was evaluated from three independent simulations.

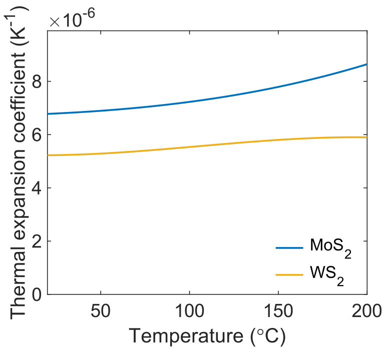

Fig. S9. TECs of MoS_2 and WS_2 monolayers as a function of temperature. From 20 °C to 200 °C, TECs of MoS_2 and WS_2 monolayers slightly increase.

Section S6. The physical range of 2D TECs

A large discrepancy by more than two orders of magnitude can be seen among the existing experimental results of TECs for the same 2D monolayer (*24*, *26*–*28*), from ~ 10^{-4} K^{-1} to ~ 10^{-7} K^{-1}, or even zero. Typical semiconductors have a TEC on the order of 10^{-6} K^{-1} (*55*). Table S3 shows the linear TECs of various materials at the room temperature, from liquids to solids. Only some liquids and polymers show highly thermal-expanding behaviors with TECs being on the order of 10^{-4} K^{-1}, the TECs of metals are on the order of 10^{-5} K^{-1}, while rigid, amorphous crystals like glass show TECs on the order of 10^{-7} K^{-1} (*55*). Through accurate experimental measurements, we show that the TECs of TMD monolayers are on the order of 10^{-6} K^{-1} and confirms the physical range of 2D TECs (from 5×10^{-6} K^{-1} to 1×10^{-5} K^{-1}). Note that the in-plane TECs measured using the three-substrate approach in this work were characterized with Raman measurements from 20 °C to 200 °C.

Table S3. Linear TECs of various materials at room temperature.

Phase	Material	α ($\times 10^{-6}$ K^{-1})
Gas		> 1000
Liquid	Ethylene glycol	209
	Water	91
Solid	Polyurethane	150
	Polystyrene	79.8
	Aluminum	22.5
	Copper	16.5
	$MoSe_2$ (this work)	8.8 ± 1.0
	WSe_2 (this work)	7.3 ± 1.1
	MoS_2 (*16*)	7.4 ± 0.5
	WS_2 (this work)	6.1 ± 0.6
	Silicon	2.6
	Fused silica	0.55
	h-BN (*40*)	-3.58 ± 0.18
	Graphene (*22*)	-8.0 ± 0.7

Section S7. Using electronegativity difference as a descriptor for 2D TMD TEC

We show the dimensionless thermochemical electronegativity difference $\Delta\chi_{\mathrm{M-X}}$ between M and X atoms (M = Mo, W; X = S, Se, Te) can serve as a descriptor to the TECs of monolayer TMDs. In this section, we explain further how $\Delta\chi_{\mathrm{M-X}}$ can well-capture the variations in the TECs of monolayer TMDs. The original concept of electronegativity was developed by Pauling (*59*), which was proposed to describe the tendency of an atom of a given element to attract shared electrons in a covalent bond with a unit of $\mathrm{eV}^{1/2}$. A higher value of the electronegativity indicates that the element attracts the shared electrons more strongly. The element-dependent electronegativity difference is thus a fundamental parameter describing the polar nature of chemical bonds, which is much more widely accessible as compared to first-principles calculated properties, like the Grüneisen parameters. Tantardini and Oganov further reformulated Pauling's electronegativity with experimental dissociation energies, which leads to the dimensionless thermochemical electronegativity (*60*). In this work, we discovered that $\Delta\chi_{\mathrm{M-X}}$ between the metal and chalcogen atoms is a fundamental parameter describing the polar nature of M-X bonds, which can be used to describe mechanical and thermophysical properties (*54*, *59*, *60*). Fig. S10A shows the relationship between in-plane stiffness predicted using density functional theory (DFT) calculations (*35*) and electronegativity difference. With the increase of $\Delta\chi_{\mathrm{M-X}}$, the inverse of the in-plane stiffness decreases linearly. The relation indicates a larger $\Delta\chi_{\mathrm{M-X}}$ in TMD monolayers, as a result of stronger polarity between M and X atoms, leads to a stiffer material. Fig. S10B depicts the relationship between TECs and in-plane stiffness. The TEC follows a linearly decreasing trend with respect to the in-plane stiffness. The stiffer a material is, the lower its TEC is (*35*, *61*, *62*). The close relationship further confirms that the dimensionless thermochemical electronegativity difference $\Delta\chi_{\mathrm{M-X}}$ can well-capture the polar nature of M-X bonds and thus serve as a descriptor to understand TECs of 2D TMD. Moreover, $\Delta\chi_{\mathrm{M-X}}$ enables a rapid estimation of unknown TECs of other 2D TMD in practical applications.

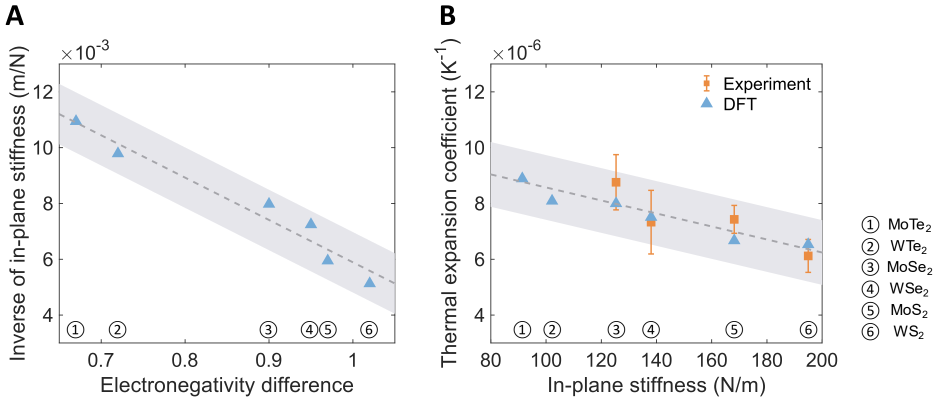

Fig. S10. The relationship among in-plane stiffness, electronegativity difference, and TECs. (**A**) The relationship between in-plane stiffness and electronegativity difference. With the increase of electronegativity difference, the inverse of the in-plane stiffness decreases linearly. (**B**) The relationship between TECs and in-plane stiffness. The TEC follows a linearly decreasing trend with respect to the in-plane stiffness. The stiffer a material is, the lower its TEC is. Dashed line: linear fitting. Grey region: the uncertainty band of one standard deviation for linear fitting.
